# Magnetic resonance colonography with intestine-absorbable nanoparticle contrast agents in evaluation of colorectal inflammation

**DOI:** 10.1007/s00330-020-07609-8

**Published:** 2021-01-06

**Authors:** Xue Dong, Jingfeng Luo, Pengxun Lan, Xiuyu Guo, Xin Zhao, Xiaoyan Wang, Fei Zhou, Qiangfeng Wang, Hong Yuan, Jihong Sun

**Affiliations:** 1 Department of Radiology, Sir Run Run Shaw Hospital, Zhejiang University School of Medicine, No. 3 East Qingchun Road, Hangzhou 310016 Zhejiang, China; 2 Department of Radiology, HwaMei Hospital, University of Chinese Academy of Sciences, Ningbo, 315000 China; 3 Department of Pharmaceutics, College of Pharmaceutical Sciences, Zhejiang University, Hangzhou, 310058 China; 4 Department of Oncology, First Affiliated Hospital, College of Medicine, Zhejiang University, Hangzhou, China; 5 Innovation Center for Minimally Invasive Techniques and Devices, Zhejiang University, Hangzhou, 310016 China

**Keywords:** Ulcerative colitis, Magnetic resonance imaging, Enema, Contrast media, Nanoparticles

## Abstract

**Objectives:**

To develop a nanoparticle-based MRI protocol based on transrectal administration of intestine-absorbable nanoparticle contrast agents to evaluate ulcerative colitis (UC).

**Methods:**

Solid lipid nanoparticles (SLNs) were synthesized by loading gadolinium diethylenetriaminepentaacetic acid (Gd-DTPA) and octadecylamine-fluorescein isothiocyanate to produce Gd-FITC-SLNs as T1 contrast agents. Twenty mice with acute UC were divided into four groups: enema with Gd-FITC-SLNs, intravenous injection of Gd-FITC-SLNs, enema with Gd-DTPA, and intravenous injection of Gd-DTPA. Five mice with chronic UC and five mice without UC underwent enema with Gd-FITC-SLNs. Axial T1- and T2-weighted MR images were obtained before and 20, 40, 60, 80,100, and 120 min after enema or intravenous injection of the contrast agent. The signal-to-noise ratios (SNRs) of the colorectal wall were measured in both groups. The MRI findings were correlated with subsequent histological confirmation.

**Results:**

At 20 min after enema with Gd-FITC-SLNs, MRI showed the following contrast enhancement pattern: acute UC > normal intestinal wall > chronic UC. A continuous enhancement effect was observed in mice with acute UC, whereas a slight continuous enhancement of the colorectal wall was observed in mice with chronic UC. The normal intestinal wall rapidly metabolized the contrast agent, and the enhancement decreased on sequential scans. There was no significant difference between the SNRs of the intestinal wall at 20 min after intravenous Gd-DTPA and transrectal Gd-FITC-SLN administration.

**Conclusions:**

Enema with Gd-FITC-SLNs may be helpful for the diagnosis and differential diagnosis of acute and chronic UC and can confer the same or better results than with intravenous Gd-DTPA.

**Key Points:**

• *Enema with Gd-FITC-SLNs may be helpful for the diagnosis and differential diagnosis of acute and chronic UC.*

• *Enema with Gd-FITC-SLNs can achieve the same or better result than that with intravenous Gd-DTPA.*

• *SLN-based MR colonography enhances the colorectal wall inflammation, based on the colonic absorption of the nanoparticle contrast agents.*

## Introduction

UC is a type of recurrent non-specific inflammation. Recently, its incidence has been increasing annually [1]. The etiology and pathogenesis of UC are poorly understood. UC lesions, which are primarily ulcers, mainly appear in the colonic mucosa, involving the rectum, distal colon, and even the entire colon in some cases [2]. UC can occur in all age groups and sexes. Presently, the disease has been listed as a modern refractory disease by the World Health Organization. With diverse clinical manifestations, patients with atypical symptoms might be ignored and misdiagnosed. The diagnosis of UC is challenging because it warrants exclusion of infectious colitis, such as bacillary dysentery, amebic dysentery, chronic schistosomiasis, intestinal tuberculosis, Crohn’s disease, ischemic colitis, and radioactive colitis, and is based on clinical manifestations, and results of colonoscopy or barium enema [3]. Therefore, medical researchers have continually explored ways of facilitating early diagnoses of UC [4].

Gd-DTPA is formerly the most commonly used positive MR contrast agent, and it is not absorbed by the digestive tract. Following intravenous administration, it is distributed throughout the body and can enter the extracellular space but not inside the cell. Gd-DTPA distribution in the lesions is only associated with blood supply, and there is no tissue targeting or specificity [5, 6]. However, Gd^3+^ in Gd-DTPA has a strong paramagnetism; thus, a higher MR signal can be obtained at relatively low concentrations (0.1–0.2 mmol/kg body weight). MR-negative contrast agents, such as superparamagnetic iron oxide, show low MR signals, which is difficult to distinguish from intestinal gas. Therefore, Gd-DTPA is more suitable for use as a contrast agent for imaging intestinal diseases. However, the structure and physicochemical properties of Gd contrast agents are very stable and cannot be absorbed by the intestinal tract. Therefore, the contrast agents for intestinal wall absorption and pathological target display can be prepared with intestine-absorbable carriers.

The application of nanotechnology can overcome many problems that plague conventional contrast agent preparations [7–9]. Nanoparticles have remarkable potential as carriers of molecular contrast agents due to their unique physicochemical properties. A new, solid, colloidal delivery system can be created by controlling the size of SLNs in the range of 50–1000 nm and encapsulating/embedding various biological molecules (drugs, DNA fragments, polypeptides, or proteins) inside its aliphatic nuclei [10, 11]. The advantages of SLNs include good biocompatibility, biodegradability, and easy absorption by the digestive tract. Approximately 50% of SLNs can be absorbed by the intestinal tract directly through the intestinal mucosa cells or extracellular space; approximately 70% of these SLNs can be transported through the lymph, while the remaining 30% can be transported directly by blood [12–14]. SLNs are metabolized through the hepatobiliary pathway and do not have any renal toxicity. The circulation time of SLNs in vivo is 12–24 h, and about 70% of the drugs are dispersed in the solid lipid skeleton. Therefore, SLNs have a slow-release effect, conducive for prolonging the examination time window. SLNs can encapsulate Gd-DTPA [15], but the application of SLN-encapsulated Gd-DTPA in intestinal MRI has not been studied. Hence, in this study, we investigated whether the prepared SLN-encapsulated Gd-DTPA (Gd-SLN), introduced via intestinal absorption, can be used for MRI-based evaluation of UC.

## Materials and methods

### Animals

Animal experiments were conducted according to protocols approved by the Institutional Committee for Animal Care, and the animals were housed according to institutional guidelines. Thirty 6–8-week-old wild-type C57/B6 female mice weighing 20–25 g were purchased from the Laboratory Animal Center. These animals were kept under a cycling 12/12-h light/dark schedule, with ad libitum access to food and water.

### Establishment of the murine UC model

Dextran sodium sulfate (DSS, 2.5%) was provided in drinking water for seven consecutive days in all mice. During these 7 days, the stool, body weight (bw), and food and water intake of the animals were monitored. The clinical UC score based on weight and stool parameters was assessed daily using a standard criterion (Table 1). One week later, if the clinical UC score of a mouse was 4 points, the murine UC model was considered to be successful and was included in the experiment.

**Table 1:** Scoring criteria of disease activity index (DAI)

Score	Decrease in weight (%)	Stool parameters
0	No	Normal
1	1–5	Loose stool
2	6–10	Mild diarrhea
3	11–15	Diarrhea
4	> 15	Hematochezia

To induce acute UC (*n* = 20 mice), the selected C57/BL mice were fed adaptively for approximately 1 week and then supplemented with 2.5% DSS (36,000–50,000 Da; 216011090; MP Biomedicals) in drinking water for 7 days.

To induce chronic UC (*n* = 5 mice), the selected C57/BL mice drank 2.5% DSS solution freely for seven consecutive days, followed by standard water for 2 weeks, and then 2% DSS, for two cycles.

Twenty mice with acute UC were randomly allocated into four study groups and were administered with the following: (i) transrectal enema with Gd-FITC-SLNs (55.6 mg/ml, *n* = 5); (ii) intravenous injection of Gd-FITC-SLNs (55.6 mg/ml, *n* = 5); (iii) transrectal enema with Gd-DTPA (9.38 mg/ml, *n* = 5); and (iv) intravenous injection of Gd-DTPA (9.38 mg/ml, *n* = 5). Five mice with chronic UC and five normal mice received a transrectal enema with Gd-FITC-SLNs.

### Contrast medium, Gd-FITC-SLNs

Gd-FITC-SLNs were prepared using the solvent diffusion method reported previously [16]. FITC-labeled octadecylamine (ODA) was synthesized as a fluorescence marker according to our previously reported protocol [16]. The Gd-DTPA entrapment efficiencies and Gd-FITC-SLN drug-loading rates were 55.8% and 16.87%, respectively. The T1 relaxivity of Gd-FITC-SLNs was 3.39 M^−1^ s^−1^, slightly lower than that of Gd-DTPA (4.22 M^−1^ s^−1^). Both the in vitro toxicity studies on Gd-FITC-SLNs, using the CCK8 assay for CT-26 cell and HT-29 cell viability, and in vivo studies using the MTD assay in C57/BL mice proved the drug to be non-toxic [17, 18].

### MRI

Food was withheld from all animals for 24 h before starting the experiment. MRI was performed using a 3.0-T MRI system (SIGNA HD, General Electric Healthcare) and a special quadrature mouse coil (inner diameter 50 mm). After placing the mice in the supine position at the center of the coil, deep anesthesia was induced by inhalation of 1.5% isoflurane (Sinopharm Bio-pharmaceutical Co., Ltd.) and oxygen gas, while allowing them to breathe spontaneously. For the enema group (transrectal enema of Gd-FITC-SLNs and Gd-DTPA), MR colonography was performed before and 20, 40, 60, 80, 100, and 120 min after the liquid intracolonic enema. First, 1-ml of indoor air was introduced into the colon and rectum using a 1-ml syringe and a 24-gauge cannula (Xindeyi Medical Instrument Co., Ltd.). A small rubber seal placed in the anus of each mouse prevented rectal leakage. After performing baseline MRI, the mice were removed from the coil for Gd-FITC-SLNs and Gd-DTPA enema using 1 ml of contrast agents, infused at a rate of 2 ml/min. The liquid enema was sustained for 20 min. A cleansing enema with water was performed before the second imaging session that was subsequently undertaken after distending the colorectum with 1 ml of room air. For the intravenous injection group (injection of Gd-FITC-SLNs and Gd-DTPA), MR colonography was performed before and 20, 40, 60, 80, 100, and 120 min after the intravenous injection of contrast agents. The protocol for baseline MRI was the same for the enema group. After MRI, the mice were removed from the coil and intravenously injected with a 0.2-ml contrast agent. Finally, the subsequent scans were undertaken in the right order. The positions of the anuses of the mice were marked on the inner surface of the coil, which ensured that they were placed in the same position during MR colonography.

After 2 pilot scans, an axial, T1-weighted, 2D spin-echo sequence (repetition time [TR] ms/echo time [TE] ms, 550/15; matrix, 256 × 256; section thickness, 1.5 mm; spacing, 0.5 mm; field of view [FOV], 6 × 4.5 cm; number of excitations [NEX] 2; and time of acquisition [TA], 3:53 min) was performed before and 20, 40, 60, 80,100, and 120 min after using the contrast agents in all mice. An axial, T2-weighted, 2D spin-echo sequence (TR/TE, 2000/80; flip angle, 90°; FOV, 70 mm; matrix, 256 × 256; slice thickness, 1 mm; spacing, 0.5 mm; NEX, 2; and TA, 3:15 min) was also performed before contrast enema or contrast injection.

### MR image analysis

The MR images were analyzed by one radiologist who was blinded to the experimental protocol. The intestinal wall is very thin, which complicated the measurements and resulted in significant errors. To overcome this challenge, we chose three consecutive and discontinuous layers of images from the colorectal wall. For each animal, each layer taken from four points in the 12, 3, 6, and 9 o’clock directions of the circular intestinal wall delineated the region of interest (ROI). The final result was the mean of all ROIs. To avoid including the surrounding structures, such as the colonic lumen, pericolonic vessels, and fat, the operator-defined ROIs in the rectal wall were kept as large as possible. The SNRs (SNR = SI_colon wall_/SI_background noise_) for T1-weighted images on the axial portion of the bowel wall were calculated and plotted over time.

### Histological and fluorescence analyses

All animals were sacrificed after MRI by cervical dislocation performed before their recovery from deep anesthesia, following which their colorectal segments were harvested. The rectal tissue was divided into two halves; half of the rectum specimens were fixed in 4% formaldehyde, embedded in paraffin, and stained with standard hematoxylin and eosin (H&E) for histological examination of inflammation. The other half was fixed with an optimal cutting temperature compound and used for fluorescence analysis to detect and localize FITC-labeled Gd-FITC-SLNs within the bowel wall; subsequently, 5-mm-thick sections of snap-frozen colon segments were analyzed under green fluorescence on a fluorescence microscope (NIKON ECLIPSE C1) and processed using the dedicated software (NIKON DS-U3). The tissue sections were then stained with DAPI for 10 min.

### Statistical analysis

For statistical analysis, the GraphPad Prism 6 (GraphPad Software, Inc.) statistical software was used. Descriptive statistical measures—mean value, standard deviation, and range—were evaluated. The data were compared with different experimental groups. The Mann–Whitney *U* test for group comparisons and Wilcoxon test for paired comparisons of quantitative measurements were performed. The comparative course of SNR with time was analyzed by multivariate ANOVA. A *p* value of < 0.05 was considered statistically significant.

## Results

### Histological and fluorescence analyses

**Fig. 1:**
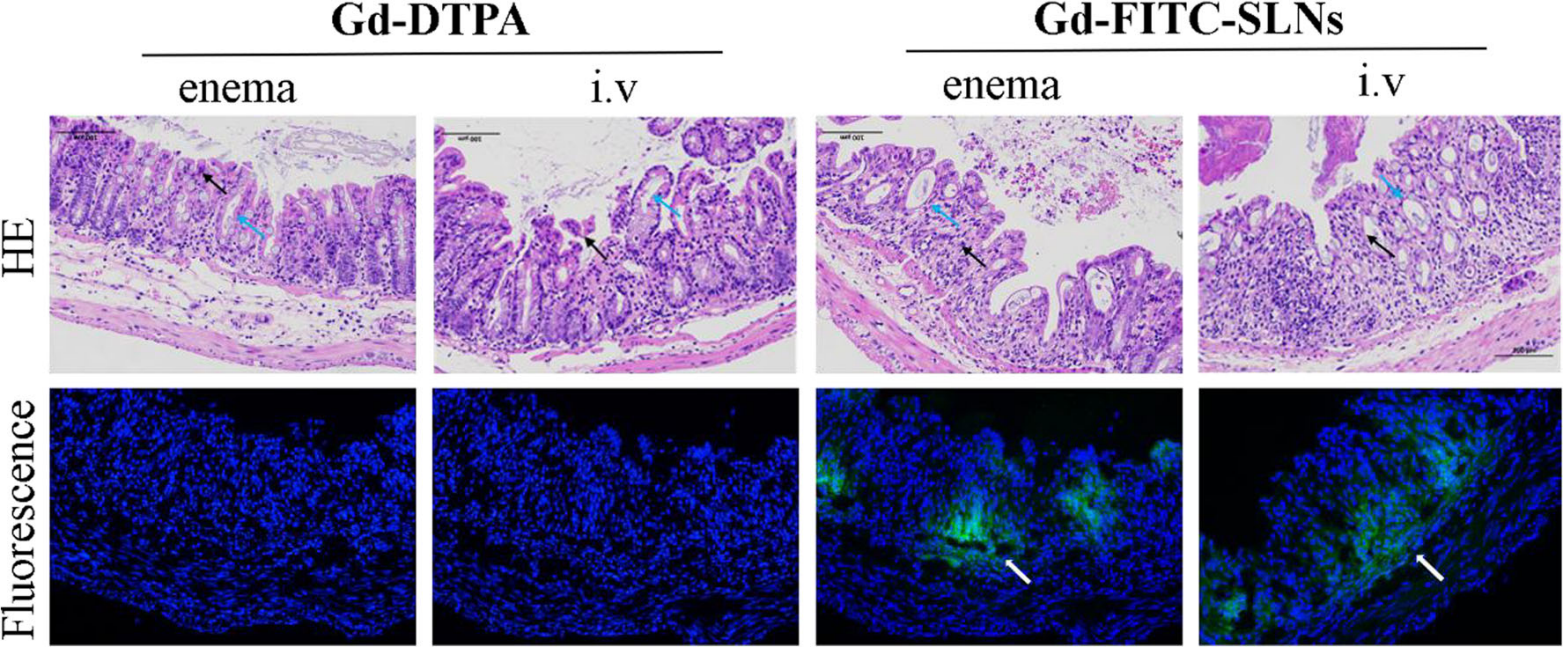
Histology of colitis in the four groups. Hematoxylin-eosin and fluorescence staining of the rectum. HE staining shows that more intestinal epithelial cells were exfoliated in the mucosal layer, replaced by proliferated connective tissue, and a few lymphocytes and neutrophils infiltrated. In the submucosal layer, mild edema was observed, and a few inflammatory cells infiltrated the submucosal layer (black arrow). Fluorescence staining showed that green fluorescent spots obviously accumulated in the colonic walls of colitic mice after using Gd-FITC-SLNs (white arrow), which is not observed when using Gd-DTPA

Histological signs of colitis were observed in all 20 mice with acute UC, with a similar degree of inflammation, which indicated successful development of the murine UC model. The four experimental groups treated with different contrast agents showed similar distributions of the degrees of inflammation (Fig. 1). Fluorescence microscopy was performed after 2 h in all four groups. Confocal fluorescence microscopy revealed that Gd-FITC-SLNs, observed as highly concentrated green fluorescent spots, accumulated in the inflamed colon wall in the enema and intravenous injection groups of Gd-FITC-SLNs (Fig. 1). However, in the intravenous injection group, Gd-FITC-SLNs were also detected in the submucosal layer.

### MR image analysis

**Fig. 2:**
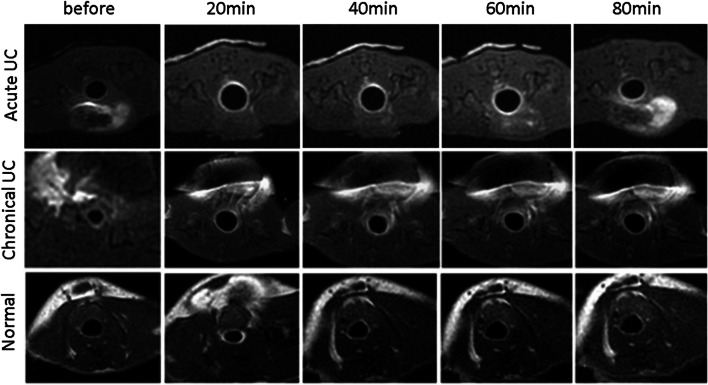
MR images of acute UC, chronic UC, and normal mice in the colorectum of mice. Dynamic MR imaging showed that the intestinal wall of acute inflammation was significantly enhanced after 20 min of retention enema with nano-contrast agent Gd-FITC-SLNs. The degree of enhancement was as follows: acute UC > normal intestinal wall > chronic UC

**Fig. 3:**
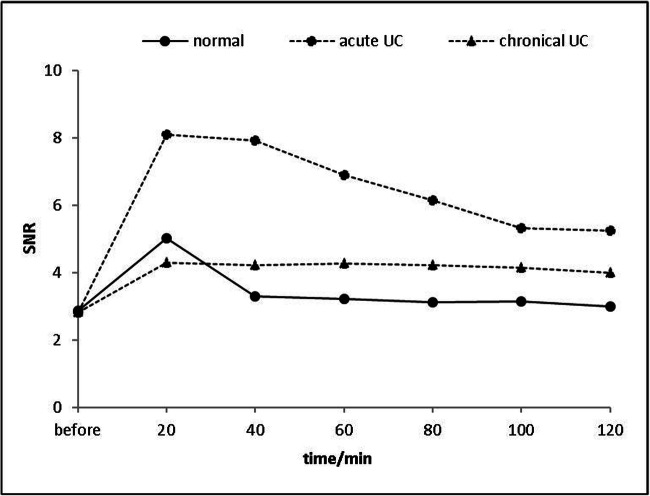
SNR of the colorectal walls before, at 20, 40, 60, 80 min, and 2 h after transrectal infusion of Gd-FITC-SLNs. After 20 min of retention enema with nano-contrast agent Gd-FITC-SLNs, dynamic MR imaging showed that the intestinal wall in acute UC was significantly enhanced and lasted for a long time. The uptake of nano-contrast agent in the normal intestinal wall was less than that in acute inflammation and the enhancement decreased faster, while the enhancement of chronic UC was weaker than that of the normal intestinal wall in 20 min

**Fig. 4:**
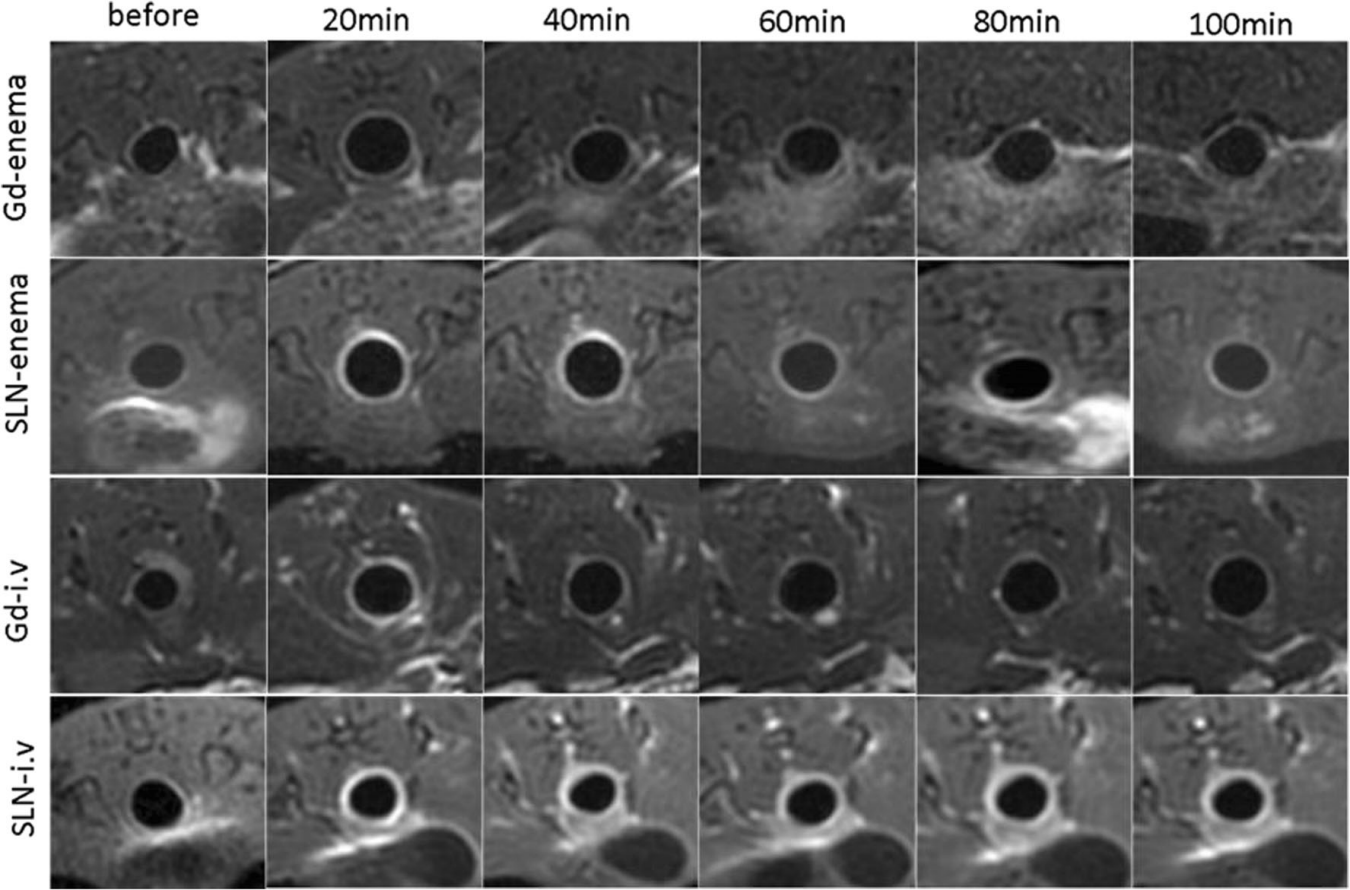
MR images of the colorectum of mice. The effect of transrectal infusion of Gd-FITC-SLNs resembled that of intravenous injection of Gd-DTPA in 20 min. Compared with intravenous injection of Gd-DTPA, the enhancement of the intestinal wall was more obvious and lasting in the inflamed bowel wall after i.v. injection of Gd-FITC-SLNs

**Fig. 5:**
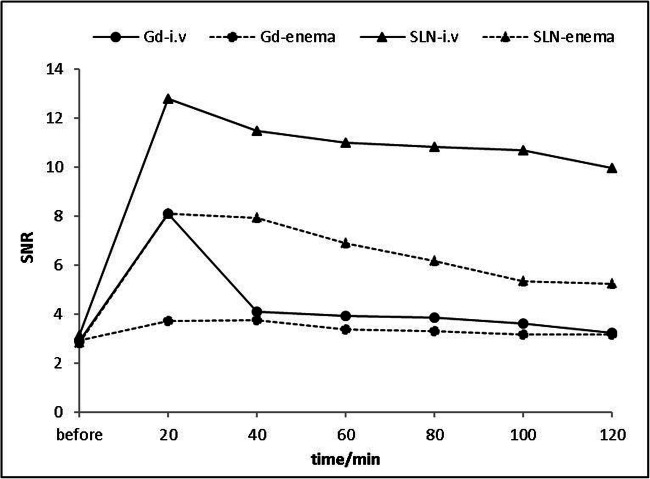
The SNR of the colorectal wall after i.v. injection and enema of Gd-FITC-SLNs and Gd-DTPA are plotted over time. To better emerge the curves, the estimated marginal means of the data are presented. The curves for the colorectal wall differ significantly after Gd-FITC-SLN enema (dotted line with triangle) as opposed to Gd-DTPA enema (dotted line with dots). There were also significant differences between intravenous Gd-FITC-SLN (solid line with triangles) and Gd-DTPA (solid line with dots). Note the prolonged contrast enhancement in the group with i.v. injection of Gd-FITC-SLN compared with that of Gd-DTPA

After 20 min of retention enema with Gd-FITC-SLNs, dynamic MRI showed colorectal wall enhancement due to nano-contrast agent absorption. The degree of enhancement presented the following pattern: acute UC > normal intestinal wall > chronic UC (Figs. 2 and 3). The colorectal wall in the acute UC group was continuously enhanced for 120 min, the normal intestinal wall showed rapid metabolization of the contrast agent, and the degree of enhancement decreased with time, while in the chronic UC group, the colorectal wall only showed slight enhancement. After 20 min of enema with Gd-FITC-SLNs, the rectal wall enhancement of mice with acute UC was similar to those administered with intravenous Gd-DTPA, but the enhancement time was longer with enema than with intravenous injection (Figs. 4 and 5). In addition, intravenous Gd-FITC-SLN injection conferred a better contrast effect.

After retention enema with Gd-FITC-SLNs, the SNR of the intestinal walls of normal, acute, and chronic UC mice differed significantly at all time points (Table 2). The eventual course of SNR differed significantly across the three degrees of inflammation (*p* < 0.01). Furthermore, in the colon wall of normal mice, the SNR at each time point did not differ significantly before and after 40 min of enema (*p* > 0.05). The intestinal wall of mice with chronic UC was slightly enhanced after 20 min of enema but showed no significant difference in SNRs between the time points after enema. In the colon wall of mice with acute UC, the SNRs were significantly higher at 120 min than that before the enema (2.81 ± 0.41 vs. 5.24 ± 0.65; *p* < 0.01).

**Table 2:** SNR of the colorectal wall: Different degrees of inflammation at various times after enema application of Gd-FITC-SLNs

SNR					
Type of mouse					
Contrast agent	Time	Normal	Acute UC	Chronic UC	*p*
	Before	2.87 ± 0.39	2.81 ± 0.41	2.82 ± 0.41	0.39
	20	5.02 ± 0.63	8.09 ± 1.71	4.29 ± 0.55	< 0.01*^‡ #^
	40	3.29 ± 0.32	7.92 ± 1.60	4.20 ± 0.49	< 0.01*^‡ #^
Gd-FITC-SLNs	60	3.21 ± 0.38	6.88 ± 1.17	4.26 ± 0.36	< 0.01*^‡ #^
	80	3.11 ± 0.45	6.15 ± 0.98	4.21 ± 0.40	< 0.01*^‡ #^
	100	3.14 ± 0.34	5.33 ± 0.59	4.14 ± 0.29	< 0.05*^‡ #^
	120	3.00 ± 0.51	5.24 ± 0.65	3.99 ± 0.57	< 0.01*^‡ #^

*SNR*, signal-to-noise ratio; *UC*, ulcerative colitis

*Significant differences between acute and chronic inflamed colon wall (*p* < 0.05)

^‡^Significant differences between normal and chronic inflamed colon wall (*p* < 0.05)

^#^Significant differences between normal and acute inflamed colon wall (*p* < 0.05)

Table 3 summarizes the SNRs of the rectal walls observed on T1-weighted images acquired after transrectal enema with Gd-FITC-SLNs, intravenous injection of Gd-FITC-SLNs, transrectal enema with Gd-DTPA, and intravenous injection of Gd-DTPA. No significant differences were found in the SNRs of the rectal wall among the four groups before intravenous injection or enema with Gd-FITC-SLNs and Gd-DTPA.

**Table 3:** Signal-to-noise ratio of the colorectal wall which had a different route of administration at various times after application of Gd-DTPA and Gd-FITC-SLNs

SNR				
Route of administration	Contrast agent			
	Time	Gd-DTPA	Gd-FITC-SLNs	*p*
i.v	Before	2.90 ± 0.40	3.12 ± 0.64	0.07
	20 min	8.09 ± 1.21	12.8 ± 2.87	< 0.01
	40 min	4.09 ± 0.72	11.45 ± 3.16	< 0.01
	60 min	3.92 ± 0.77	10.99 ± 3.07	< 0.01
	80 min	3.85 ± 0.75	10.82 ± 3.03	< 0.01
	100 min	3.61 ± 0.60	10.67 ± 2.88	< 0.01
	120 min	3.24 ± 0.46	9.96 ± 2.32	< 0.01
Enema	Before	2.92 ± 0.23	2.81 ± 0.41	0.36
	20 min	3.72 ± 0.50	8.09 ± 1.71	< 0.01
	40 min	3.74 ± 0.45	7.92 ± 1.60	< 0.01
	60 min	3.38 ± 0.42	6.88 ± 1.17	< 0.01
	80 min	3.29 ± 0.33	6.15 ± 0.98	< 0.01
	100 min	3.16 ± 0.54	5.33 ± 0.59	< 0.01
	120 min	3.14 ± 0.34	5.24 ± 0.65	< 0.001

*Gd-DTPA*, gadofluorine diethylenetriaminepentaacetic acid; *SNR*, signal-to-noise ratio; *i.v.*, intravenous

The SNRs of the rectal wall showed statistically significant differences between the time points after enema with Gd-FITC-SLNs and Gd-DTPA, especially after 20 and 40 min (3.72 ± 0.50 vs. 8.09 ± 1.71, *p* < 0.01; 3.74 ± 0.45 vs. 7.92 ± 1.60, *p* < 0.01, respectively). In addition, there was no significant difference between the SNRs of the intestinal wall at 20 min after intravenous Gd-DTPA injection and transrectal enema with Gd-FITC-SLNs (8.09 ± 1.21 vs. 8.09 ± 1.71, *p* > 0.05; Table 4). However, no significant differences were found in SNRs of the rectal wall at any time after Gd-DTPA enema.

**Table 4:** Signal-to-noise ratio of the colorectal wall between i.v. Gd-DTPA and transrectal enema of Gd-FITC-SLN

Time	SNR		*p*
	i.v. Gd-DTPA	Transrectal enema of Gd-FITC-SLN	
Before	2.90 ± 0.40	2.81 ± 0.41	0.18
20	8.09 ± 1.21	8.09 ± 1.71	0.99
40	4.09 ± 0.72	7.92 ± 1.60	< 0.05*
60	3.92 ± 0.77	6.88 ± 1.17	< 0.05*
80	3.85 ± 0.75	6.15 ± 0.98	< 0.01*
100	3.61 ± 0.60	5.33 ± 0.59	< 0.05*
120	3.24 ± 0.46	5.24 ± 0.65	< 0.01*

*Significant differences between i.v. Gd-DTPA and transrectal enema of Gd-FITC-SLN

After intravenous contrast injection, the SNR of the rectal wall showed statistically significant differences at all time points after the intravenous Gd-DTPA injection than that after intravenous Gd-FITC-SLN injection, especially after 40 min (Table 3). In addition, there were significant statistical differences in the eventual courses of the SNR between the two different contrasts (*p* < 0.01). For intravenous Gd-DTPA injection, the SNR at 20 min had increased, but the SNR before and 120 min after contrast injection did not differ significantly (2.90 ± 0.40 vs. 3.24 ± 0.46, *p* > 0.05). After intravenous Gd-FITC-SLN injection, the SNRs were significantly higher at all time points after the contrast agent injection than those before injection. After Gd-FITC-SLN intravenous infusion, the inflammatory bowel wall showed obvious enhancement at all time points; the degree of enhancement was greater than that with intravenous Gd-DTPA injection. The results show that MRI with Gd-FITC-SLNs, administered by intravenous injection and enema, can be used to diagnose UC.

## Discussion

Radiological imaging is an important non-invasive method for the diagnosis of UC. Compared with CT, MRI has several advantages, such as high-resolution imaging, detailed soft tissue background, and no need for ionizing radiation [19–21]. With a higher spatial resolution and multi-parameter imaging, MRI is suitable for the early diagnosis of UC and assessment of disease activity [22]. Magnetic resonance enterography is the most commonly used technique in UC, which uses air or water enema to dilate the colorectal lumen followed by intravenous injection of Gd-DTPA to enhance the colorectal wall [23]. However, intravenous Gd-DTPA is confined to the extracellular space and is not selectively distributed throughout the body [24–26]. Recent studies have shown that Gd may be deposited in the brain and its use may increase the risk of renal fibrosis; therefore, gadolinium is contraindicated in patients with renal insufficiency [27]. Compared with intravenous injection, oral or enema of contrast agent administration can significantly reduce side effects, which indicates the importance of these routes of administration.

With the development of nanotechnology, new molecular probes have been used for targeted imaging of lesions and surrounding tissues. SLNs are colloidal particles composed of proteins, dendrimers, or liposomes. They are 50–1000 nm in size and have the advantages of biocompatibility, biodegradability, and easy absorption by the digestive tract. Although most contrast agents require intravenous injection, SLNs are mainly absorbed through the intestine. When taken orally, the proportion of gastric absorption to total absorption is less than 5% [28]. Within a given concentration range, approximately 50% of SLNs can be absorbed directly through the intestinal mucosa or intercellular space in a linear relationship; about 70% of the absorbed SLNs enter the circulation through the lymphatic system [13]. Through the lymphatic transport, SLNs can escape the first-pass metabolism in the liver, which improves absorption and its efficacy. Animal studies have confirmed that SLNs can improve drug delivery and absorption in the intestinal epithelial cells [29–31]. Although the rectal mucosa can absorb water, electrolyte, and some drugs, there is no MRI technology based on the colorectal absorption of contrast agents for UC diagnosis. With SLNs as the carrier, Gd-DTPA was loaded to synthesize Gd-SLNs as an MR colon contrast agent for direct absorption by the colorectal wall [16]. In addition, Gd-DTPA and FITC produce Gd-FITC-SLNs for T1 contrast agent and histological confirmation of MR findings. Our MRI contrast can diagnose UC by local enema, thus reducing the content of Gd in the whole body. SLNs can also be loaded with therapeutic drugs, which may have the utility for further targeted treatment of UC.

Our results showed that Gd-FITC-SLNs, a novel nano-contrast agent, can significantly enhance the inflammatory bowel wall after administration by enema. The degree of enhancement is similar to that obtained following intravenous Gd-DTPA injection, the most commonly used method in clinics. The effect of enema can last for more than 120 min, much longer than that for intravenous Gd-DTPA injection. In addition, dynamic scanning can help to diagnose and differentially diagnose acute and chronic UC according to observed the enhancement degree and changes in the colon wall after Gd-FITC-SLN enema. The degree of enhancement showed the following pattern: acute UC > normal mice > chronic UC, and the colon wall of mice with acute UC was continuously enhanced for 120 min. The agent was rapidly metabolized in normal intestinal walls and the enhancement degree decreased, while the colorectal wall in chronic UC was only slightly enhanced. The inflammatory bowel wall can also be enhanced by intravenous Gd-FITC-SLN injection, and its enhancement effects and duration are longer than those with Gd-DTPA. Therefore, enema with Gd-FITC-SLN can achieve or even improve the effect of Gd-DTPA when compared with its intravenous injection.

This novel SLN-mediated enema imaging method has several significant advantages: (i) contrast-enhanced MRI of the inflammatory intestinal wall can be achieved with a high concentration of Gd-DTPA-carrying SLNs absorbed by the intestinal wall, which can avoid intravenous Gd-DTPA injections, thus reducing its systemic amount in the whole body; (ii) it is helpful for the diagnosis and differential diagnosis of acute and chronic UC; and (iii) it provides a theoretical basis for further local treatment of UC with targeted Gd-SLNs by conjugating target-specific ligands into SLNs. When the targeted Gd-SLN is loaded with anti-inflammatory drugs, such as *Tripterygium wilfordii*, the nanomaterial-based integration of diagnosis and treatment of UC can be achieved.

We previously showed significant enhancement of tumor site after transrectal infusion of Gd-FITC-SLNs in mice with AOM/DSS-induced colorectal high-grade intraepithelial neoplasia [32]. However, the potential mechanisms underlying of enhanced absorption of Gd-FITC-SLNs in the inflamed bowel wall with transrectal infusion are unclear. We tried to explain the underlying mechanisms of imaging using Gd-FITC-SLNs. An important factor in the pathogenesis of IBD is the accumulation of phagocytes such as macrophages and dendritic cells in the intestinal wall. Some studies have shown the destruction of that the epithelial cell-to-cell junction protein in the intestinal wall of patients with IBD [33–38]. Therefore, the phagocytosis of macrophages and the destruction of intercellular linkers may be the main mechanism by which nanomaterials enter the intercellular space. First, SLNs can specifically adhere to the mucosa, be absorbed by the epithelial cells, and spread to the extracellular space. Second, because inflammation causes changes in the small structure of the intestinal wall, SLNs may aggregate, in the form of micelles, inside the macrophages infiltrating the inflamed bowel wall. Our confocal fluorescence microscopy analysis also showed that FITC-labeled contrast agents (Gd-FITC-SLNs) accumulated in the lamina propria mucosa of the bowel. Because Gd-FITC-SLNs have longer half-lives than Gd-DTPA, compared to the intravenous Gd-DTPA injection, prolonged enhancement in the inflamed bowel wall after intravenous Gd-FITC-SLN injection could be partially explained by the blood-pool effects in dilated vessels, which is caused by their general hyperemic reaction to an inflammatory stimulus.

Our study has a few limitations. First, the mechanism underlying Gd-FITC-SLNs absorption by the inflammatory intestinal wall is based on our hypothesis; further research is needed to confirm this hypothesis. Second, no further study was conducted on the different enhancement methods of Gd-FITC-SLNs in mice with acute and chronic UC. Third, the sample size per experimental group was small (*n* = 5). Our results need validation by further studies with a larger sample size. Fourth, because of the presence of air in the intestinal cavity, the magnetic susceptibility artifact will be produced between the gas and the surrounding intestinal wall tissue, which reduces the image clarity. Future studies can reduce susceptibility artifacts by oral administration of low-dose paramagnetic contrast agents. Lastly, like other animal models, DSS-induced UC models do not fully reproduce human diseases. Animal models are thus needed, such as genetic models of UC, which can accurately reproduce human diseases [39].

In conclusion, we developed a method for MRI-based evaluation of UC using a multifunctional nanoparticle contrast agent that can be administered by enema; the method is based on the absorption of nanoparticle contrast agents by the colorectal wall. SLNs administered by enema for performing MRI may open new modalities for the diagnosis and differential diagnosis of acute and chronic UC and may achieve the same or better results than MRI with intravenous Gd-DTPA injections. In addition, this novel type of MRI technique may guide in the targeted local therapy of UC once the MRI-detectable nanoparticles are loaded with anti-inflammatory therapeutics.

## Abbreviations

FITC: Octadecylamine-fluorescein isothiocyanate
Gd-DTPA: Gadolinium diethylenetriaminepentaacetic acid
MRI: Magnetic resonance imaging
ROI: Region of interest
SLNs: Solid lipid nanoparticles
SNR: Signal-to-noise ratio
UC: Ulcerative colitis
